# Myopia in Beagles in a Family of 12 Individuals

**DOI:** 10.3390/ani15162342

**Published:** 2025-08-11

**Authors:** Juliana Giselbrecht, Barbara Nell

**Affiliations:** Department of Companion Animals and Horses, University of Veterinary Medicine, 1210 Vienna, Austria

**Keywords:** dog, nearsighted, night vision impairment, retinoscopy, refractive error

## Abstract

This case report investigated the cause of visual impairment at night in Beagle dogs in a family of 12 individuals. Four related adult male Beagles with impaired night vision and eight related Beagles (three females, five males) underwent a complete ophthalmological examination at the ophthalmology service. Electroretinography was performed on four dogs with impaired night vision after dark adaptation to evaluate retinal function. Retinoscopy was performed in 12 dogs in a standing or sitting position to assess refraction. Axial globe measurements were conducted using B-scan ultrasonography in nine dogs. In total, twelve adult Beagles (nine males, three females) from four generations were evaluated, with nine dogs showing impaired night vision. Ophthalmic examinations revealed no abnormalities that could explain the visual impairment. Electroretinography showed normal retinal function. In total, 83.3% (10/12) of the dogs were myopic, with refractive errors ranging from −1.25 to −6.25 diopters (D). All dogs with night vision impairment were significantly more myopic (median: −4.88 D) than those without impairment (median: −1.25 D). In two myopic dogs, the insertion of contact lenses improved navigation in the dark maze test. Myopic dogs showed a significantly greater vitreous body depth (10.1 mm; range 9.7–10.3 mm) compared to emmetropic dogs (9.5 mm; range: 9.4–9.6 mm). These findings suggest that in dogs with night vision impairment, retinoscopy should be included in the ophthalmological exam to exclude myopia as a potential cause. Further research is needed to determine the cause of myopia in the tested Beagles and to investigate possible genetic factors.

## Simple Summary

This case report investigated the cause of night vision problems in a family of 12 related Beagle dogs. Nine dogs showed signs of impaired vision in low-light conditions. Each dog underwent a complete eye examination. Electroretinography was normal in affected dogs, suggesting normal retinal function. Retinoscopy revealed that 10 of the 12 dogs were nearsighted (myopic), with varying degrees of myopia. Dogs with night vision problems were significantly more myopic (median: −4.88 diopters) than those without (median: −1.25 diopters). These findings suggest that myopia could be a contributing factor to night vision impairment in dogs. The study recommends including retinoscopy in eye exams when night vision issues are suspected. Further research is needed to investigate the underlying cause of myopia in these Beagles, including potential genetic influences.

## 1. Introduction

Reduced night vision or night blindness in dogs, also known as nyctalopia, can be caused by various factors, typically involving genetic disorders that affect the development or function of the retina, particularly the rod cells responsible for vision in low-light conditions. One genetic condition is progressive rod-cone degeneration (PRCD), an autosomal recessive form of late-onset progressive retinal atrophy (PRA) affecting various dog breeds. It leads to bilateral retinal thinning, primarily impacting rod photoreceptors, resulting in night blindness and progressing to total blindness. Another condition is Congenital Stationary Night Blindness (CSNB), a non-progressive genetic disorder. It results from defects in the transmission of signals between the photoreceptor cells in the retina and the brain, affecting breeds like Briard dogs. This specific retinopathy due to a mutation in *RPE65* has been studied in detail and demonstrates abnormal single-flash electroretinogram responses and reduced b-waves, which are only present under photopic (bright light) conditions [1]. In the Beagle, a form of non-progressive night blindness but normal day vision has been reported in a naturally occurring colony. The dogs showed normal fundi and fluorescein angiograms. The ERG revealed the absence of a dark-adapted b-wave, while the a-wave maintained a normal amplitude [2]. A few years later, a genome-wide association study in a Beagle research colony identified a 1 bp deletion in LRIT3 segregating with CSNB [3].

Furthermore, Vitamin A deficiency can lead to night blindness in dogs. Vitamin A is crucial for the production of rhodopsin, a pigment found in rod cells. Over time the deficiency can affect the overall vision and lead to complete blindness if left untreated [4,5].

Another phenomenon that is known in humans is reduced night vision due to myopia (night myopia). Uncorrected or under-corrected myopia is most likely less noticeable during the day due to bright light, which constricts the pupil and reduces retinal blur. Night myopia may also be explained by positive spherical aberration, which causes increased retinal blur in dim light when the pupil dilates. This blurring is more noticeable at night and results in myopia. Increased accommodation in response to this blur can also enhance the myopic effect [6]. In general, myopia occurs when the eyeball is elongated or the cornea is excessively curved, leading to a focus point in front of the retina. Furthermore, age-related conditions, such as nuclear sclerosis and cataract formation, can impair vision and lead to changes in refractive error [7].

This case report describes male Beagles in a family of 12 individuals, presented with a history of visual impairment at night with suspected myopia as the underlying cause.

## 2. Materials and Methods

### 2.1. Animals

Four related castrated male adult Beagle dogs (age: 6–11 years) were presented at the ophthalmological service (University of Veterinary Medicine Vienna) due to impaired night vision. In addition, eight other related Beagles were ophthalmologically examined.

Before the ophthalmological examination, a maze test consisting of a total of 5 obstacles (1 table, 3 chairs and a rubbish bin) was carried out in the examination room. The dogs were not familiar with the examination room. The obstacle course was first carried out with the light switched off (0 Lux) and then in ambient light (1.078 Lux).

### 2.2. Ophthalmological Examination

A complete ophthalmic examination was performed by an ECVO resident under supervision of an ECVO diplomate in all dogs. Each examination included slit-lamp biomicroscopy (Kowa SL-15; Kowa, Tokyo, Japan), indirect ophthalmoscopy (Keeler Vantage; Keeler Instruments Inc., Broomall, PA, USA), Schirmer tear test-1 (Teststreifen, MSD, Unterschleißheim, Germany), fluorescein staining (Fluoro Touch^®^ Ophthalmic Strips, Eickemeyer, Tuttlingen, Germany), and measurement of intraocular pressure using rebound tonometry (TonoVet, Icare, Vantaa, Finland). Chromatic pupillary light reflex (cPLR) was assessed using a cPLR device (Melan-100^®^; BioMed Vision Technologies, Ames, IA, USA) which delivers red light at 630 nm and blue light at 480 nm wavelengths. The posterior eye segment was examined following pharmacological mydriasis (Tropicamide 0.5%; Mydriaticum Agepha^®^, Senec, Slovakia).

### 2.3. Retinoscopy

Streak retinoscopy was performed with each dog in a sitting or standing position under dimmed conditions (24 Lux). The examination was first conducted by the ECVO resident and then repeated by the ECVO diplomate. Both examiners performed the assessments independently of each other. An assistant helped with the procedure. Retinoscopy was carried out undilated by use of a streak retinoscope (Heine BETA 200, Heine Optotechnik, Herrsching, Germany) and two retinoscopy bars (Luneau Ophthamologie, Chartres Cedex, France). Retinoscopy was conducted randomly in a horizontal and vertical meridian with a working distance of 67 cm (1.5 diopters, D) [8]. Emmetropia was defined as retinoscopy values between −0.5 and 0.5 D. Myopia was defined as a refractive error of more than −0.5 D.

### 2.4. Measurements of Ocular Structures

B-scan ultrasonography (Vinno 6 Vet, Vinno, Germany) was performed under topical anesthesia (Novain 0.4%^®^ eye drops, Agepha Pharma, Bratislava, Slovakia) on both eyes in nine dogs. For accurate measurements of ocular structures, the lens was centered, the lens capsule was clearly delineated, and the optic nerve was visualized in a central position. Axial length (AL), anterior chamber depth (ACD), lens thickness (LT), and vitreous chamber depth (VCD) were measured.

### 2.5. Electroretinography

Electroretinography was performed on both eyes of four dogs with impaired night vision under general anesthesia (premedication: butorphanol 0.2 mg/kg intravenous (IV), medetomidine 10 µg/kg IV; induction: propofol 2–5 mg/kg IV to effect; maintenance: isoflurane) after 30 min of dark adaptation with pharmacologic mydriasis (Tropicamide 0.5%; Mydriaticum Agepha^®^, Senec, Slovakia) following the same protocol for all dogs. A contact lens electrode (RM Electrode™, LKC Technologies, Gaithersburg, MD, USA) and a platinum ground electrode were placed at the occiput, and a platinum reference electrode was placed three centimeters (cm) behind the lateral canthus (Grass; Natus Neurology, Middleton, WI, USA) using a handheld ERG unit (ReteVet, LKC Technologies, Gaithersburg, MD, USA). An international society for clinical electrophysiology of vision (ISCEV)-based ERG protocol based on settings of published guidelines for ERGs in dogs was used [9].

### 2.6. Gene Test

Six Beagles (B1, B2, C1, C2, D4, D5) were tested for congenital stationary night blindness (CSNB; LRIT-3 gene; GENOMIA genetic laboratory, Plzeň, Czech Republic) using whole blood (B2, C1, C2, D4, D5) or a saliva swab (B1).

### 2.7. Insertion of Human Contact Lenses

In two dogs (B1, D5), myopia was verified using corrective human contact lenses (TopVue Plus hydrogel lens, Alensa s.r.o., Praque, Czech Republic). These lenses feature a base curve of 8.6 mm and a diameter of 14.3 mm. Contact lenses were applied under local anesthesia in both animals.

### 2.8. Data Analysis

Data collection and analysis were performed using Microsoft Excel (Microsoft Corporation 2021, Redmond, WA, USA) and the R statistical language (version 4.1.2; R Core Team, 2020; R Foundation for Statistical Computing, Vienna, Austria). The normality of data distribution was determined by the Shapiro–Wilk test and histogram. Due to normally distributed data, *t* test was used for statistical analysis. Results with a *p*-value < 0.05 were considered statistically significant. Due to the lack of data, mode of inheritance could not be evaluated.

## 3. Results

### 3.1. Animals

**Figure 1:**
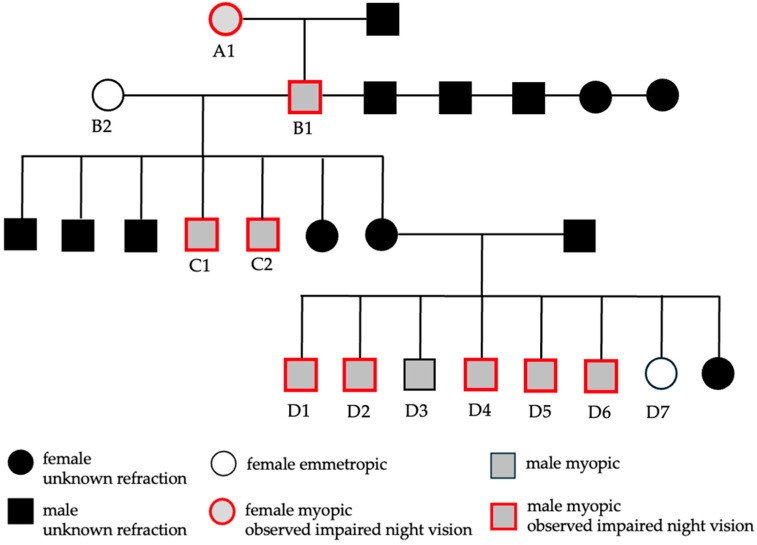
The pedigree of the Beagle colony, including dogs from four generations (A–D). Each dog is labeled with a consecutive number. Dogs with reduced vision at night are circled in red, while those that could not be examined are marked in black.

A total of twelve adult Beagles (three females and nine males) aged 6–16 years were included in this study, representing four generations (Figure 1). The four male Beagles from two generations presented with impaired night vision, showed signs of fear in the dark, and failed to recognize their owners at night. From the groups of the eight related animals, one female and four male Beagles had similar night vision impairment according to their owners. The remaining three Beagles (two females and one male) were reported to have normal vision.

### 3.2. Ophthalmological Examination

All dogs showed a positive menace response and dazzle reflex. The pupillary light reflexes were prompt and complete in ten dogs, except for two dogs with iris atrophy. The chromatic assessment of the PLR was unremarkable in all dogs, except for the two dogs with iris atrophy and incomplete PLR responses. All dogs navigated the obstacle course without difficulty under bright light. In dim light, three dogs moved confidently (B2, D3, D7), and four dogs showed uncertain behavior in the form of slow and cautious movements while avoiding obstacles (A1, D1, D2, D6), whereas five dogs bumped into objects (B1, C1, C2, D4, D5) (Table 1). All dogs showed a breed-typical macroblepharon. Tear production (right eye: 19.7 ± 1.8 mm/min; left eye: 19.6 ± 1.2 m/min) and intraocular pressure (right eye: 15.4 ± 1.8 mmHg; left eye: 16.2 ± 1.6 mmHg) were within the reference range in all dogs. The fluorescein test was negative in all dogs. One dog had an iris cyst (A1), while all other dogs showed no abnormalities in the anterior chamber of the eye. Five dogs had nuclear sclerosis (A1, B1, B2 (mild stage), C1, C2 (early stage)), and three dogs had an incipient equatorial cortical anterior cataract (A1, B1, D2). The remaining dogs showed no abnormalities of the lens (D1, D3, D4, D5, D6). Funduscopic examination was unremarkable in all dogs.

### 3.3. Retinoscopy

**Figure 2:**
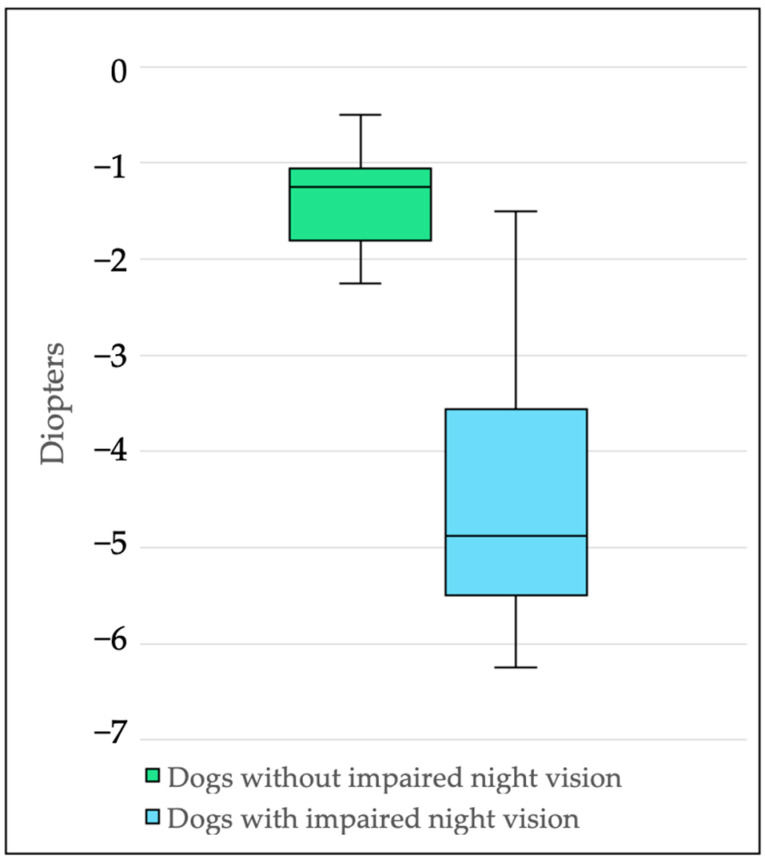
The boxplots illustrate the distribution of diopter values (D) measured in dogs using retinoscopy. Dogs that ran into obstacles in the dark were significantly more myopic (median: −4.88, range: −1.5 D–−6.25 D, *p* < 0.001) than dogs that successfully completed the obstacle course both in light and dark conditions (median: −1.25, range: −0.5 D–−2.25 D). The box represents the interquartile range (IQR), the line in the middle indicates the median, and the whiskers show the spread of the data.

All 12 dogs underwent retinoscopy to determine the refractive status. In total, 10/12 dogs (83.3%) were found to be myopic, with refractive errors ranging from −1.25 to −6.25 D. Two dogs (B2, D7) showed emmetropia (−0.25 to −0.5 D). More than half of the myopic dogs (6/10; 60.0%) showed either incipient cataracts or nuclear sclerosis (Table 1). Dogs with impaired night vision (7/12; 58.3%) were significantly more myopic (median: −4.88, range: −1.5 D to −6.25 D, *p* < 0.001) than dogs without visual impairment at night (5/12; 41.7%) (median: −1.25, range: −0.5 D to −2.25 D) (Figure 2). The findings from both examiners who conducted the retinoscopy were summarized using a mean value, with the difference between the two examiners not exceeding 0.5 D.

### 3.4. Measurements of Ocular Structures

The eye parameters were measured in 9/12 dogs using B-scan ultrasound. The average AL in emmetropic dogs was 20.7 mm (range: 20.4–20.9 mm), which was in a similar range as the globe length of dogs with myopia, measuring 21.3 mm (range: 20.9–21.6 mm). Anterior chamber depth (emmetropic dogs: 3.6 mm; range: 3.3–3.9 mm/myopic dogs: 3.8 mm; range: 3.6–3.9 mm) and LT (emmetropic dogs: 7.6 mm; range: 7.5–7.6 mm/myopic dogs: 7.5 mm; range: 7.4–7.6 mm). Myopic dogs showed a significantly greater vitreous body depth (10.1 mm; range 9.7–10.3 mm) compared to emmetropic dogs (9.5 mm; range: 9.4–9.6 mm). The results of the individual dogs are shown in Table 2.

### 3.5. Electroretinography

Electroretinography was performed on four dogs (B1, D1, D5, D6) with impaired night vision. Electroretinography results showed normal a- and b-waves in three of the four dogs, indicating normal retinal function. In one dog, reduced amplitudes were observed, likely due to incomplete mydriasis affecting the accuracy of the retinal response measurements (Table 3). The flicker response was assessed using the ISCEV 6-step protocol, but only low light intensity was applied, resulting in relatively low flicker amplitudes (mean amplitude: 33.9 µV. In one dog (D5), the flicker response was also assessed at a higher light intensity, resulting in a significantly higher amplitude (68.7 µV) compared to low light intensity (46.1 µV).

### 3.6. Gene Test

Six dogs (B1, C1, C2, D4, D5, whole blood sample; B2, saliva swab sample) were found to be negative for LRIT-3 CSNB-associated genetic mutation (N/N).

### 3.7. Insertion of Human Contact Lenses

Due to the small diameter of the contact lenses (14.3 mm) compared to the larger corneal surface area in dogs, proper centration of the lenses could not be achieved. Both dogs successfully completed a maze test conducted under dark conditions without colliding with obstacles. One dog (B1) wore the lenses continuously for one week. In the other dog (D5), the exact wearing time could not be determined, as the lenses were no longer present at the follow-up after one week. According to the owner, dog B1 showed clear signs of improved vision while wearing the lenses. The dog more effectively detected and avoided obstacles during agility training.

## 4. Discussion

This is the first case report documenting night vision impairment in a population of related Beagle dogs, which appears to be caused by underlying myopia. Neither the ten myopic nor the two emmetropic dogs showed any ophthalmic changes, except for early or mild nuclear sclerosis in five and incipient cataracts in three dogs.

Myopia has been observed in dogs and can result from a combination of genetic factors, ocular development, and external influences. In this study, 10/12 (83.3%) of the Beagles were myopic. Of the ten dogs (one female and nine males), nine dogs showed reduced vision or uncertainty at night. A correlation was found between the severity of myopia and the visual impairment in the dark. In humans, a phenomenon called night myopia is known. People with night myopia struggle to see distant objects clearly in dim light, even though their vision may be relatively normal during the day [10]. With higher levels of myopia, light is focused further in front of the retina, resulting in greater blurriness of distant objects. This effect becomes more pronounced in low-light conditions, as the pupils dilate and the incoming light in turn enhances spherical aberrations. In a study evaluating the refractive value using retinoscopy in healthy Beagles, the majority of the dogs were found to be emmetropic. However, it was noted that in Beagles with myopia, the condition tended to worsen with age. Those aged eight to nine years exhibited significantly more advanced myopia (−0.29 ± 0.82 D (range: −2.0 to 1.0 D)) than Beagles aged three to six years (0.26 ± 0.84 D (range: −2.0 to 2.0 D)). The authors made the sclerosis of the lens nucleus responsible for myopia, affecting its accommodation [11]. No relationship between age and myopia was noted in this study; nevertheless, five of the ten myopic Beagles with nuclear sclerosis and one female (B2) with nuclear sclerosis were emmetropic, and one 16-year-old female (A1) showed an incipient cataract, which could be partially responsible for the myopia. In the dark, this dog had no problems passing the obstacle course. A recent study revealed a slight, non-significant increase in myopia with higher grades of nuclear sclerosis [7]. Another study showed that aging dogs, particularly those with moderate to advanced nuclear sclerosis, develop a significant myopic shift, with refractive errors around −2.00 to −3.00 D in two individuals aged 10 and 14 years [12]. These changes mirror visual alterations seen in humans with nuclear sclerosis, including myopia, reduced contrast sensitivity, increased glare, and diminished visual acuity due to lens hardening. In the Beagle population included in this study, no correlation could be established between age and the severity of visual impairment at night. Additionally, it cannot be determined whether the reported deficits in night vision were present from an early age, as the dogs were not under the care of their current owners since puppyhood, and no reliable historical information is available regarding their early visual behavior. In this study, visual impairment at night was observed predominantly in male Beagles, with myopia reaching up to −6.25 D. To date, there is no clear evidence that myopia is more common in males than in females. In most studies on myopia in animals, gender does not play a significant role. Nevertheless, it should be noted that male Beagles were generally overrepresented across the four generations included in this study (Figure 1), which may have introduced a sex-related bias.

To identify the causes of myopia in the study population, a B-scan ultrasound was performed to determine the axial globe dimensions. B-scan ultrasound was used as A-scan devices were not available. Although this method has a potential risk of inaccuracy if the ultrasound beam is not aligned with the ocular axis, previous studies show no statistically significant difference between the axis measurements of the two methods [13,14]. In the present study myopic dogs showed a significantly greater average vitreous body depth (10.1 mm) compared to emmetropic dogs (9.5 mm). Myopic dogs also showed a longer average axial globe length (21.3 mm) compared to emmetropic dogs (20.7 mm); however, the results were not statistically significant. Unfortunately, dogs with significant myopia (C1, C2) were not available for ultrasound examination. This limitation prevented the collection of comprehensive data on their ocular measurements, compromising the understanding of the relationship between myopia and ocular structure dimensions. In a study involving various dog breeds, the mean axial length was 20.9 mm among 50 eyes [15]. In a human study myopic eyes became significantly longer with increasing refractive error (0.35 mm/D) [16]. However, Murphy et al. [15] found no correlation between the size of the canine eye and its refractive state. Myopia was likely of lenticular origin, as there were no significant differences in corneal curvature or axial length between myopic and nonmyopic German Shepherd dogs. Additionally, no linear relationship between the ocular components and refractive error was observed [15]. In this study, corneal topography using keratometry was not assessed, and the measurement of corneal curvature using retinoscopy is limited in accuracy. In a previous study, spontaneous myopia was most common in Toy Poodles (63.9%), followed by English Springer Spaniels (36.4%) and Collies (35.7%). However, the study found no significant differences in axial lengths or vitreous chamber depths between myopic and non-myopic dogs. Myopic dogs had a steeper anterior lens and greater lens power compared to non-myopic dogs. The cause of the myopia appeared to be lens-related, rather than axial elongation [17]. In the English Springer Spaniel, hereditary lenticular myopia was identified [18]. Mutti et al. [19] investigated myopia in Labrador retrievers and its relationship to ocular components. Out of 75 dogs tested, 14.7% exhibited myopia in at least one eye, with 8.0% being myopic in both eyes. Refractive errors ranged from 3.50 to −5.00 D. A significant negative correlation was found between refractive error and vitreous chamber depth, with myopic dogs having a longer vitreous chamber (10.87 ± 0.34 mm) compared to non-myopic dogs (10.02 ± 0.40 mm). These findings suggest that myopia in Labrador retrievers is similar to human myopia, primarily caused by vitreous chamber elongation [19]. Another previous study associated myopia in Labrador Retrievers with an elongated vitreous chamber, suggesting a potential genetic basis for the condition [20]. All Beagles included in the study were from the same genetic line, suggesting a potential hereditary component. A pedigree analysis could not be conducted due to lack of data; nevertheless a genetic link is suspected.

The potential for correcting myopia in dogs using soft contact lenses designed for humans was demonstrated. Both dogs in this trial showed moderate bilateral myopia, with refractive errors of −4.0 to −4.25 D (B2) and −3.5 to −3.75 D (D5). Although the contact lenses used were not designed for canine anatomy and a proper centration of the lenses was not achieved, they were tolerated without complications and provided improvement in visual function. Ofri et al. [21] evaluated eight dogs completing three retrieval trials over a distance of 137.2 m while fitted with soft contact lenses of different dioptric powers: 0 D, +1.50 D, and +3.00 D. The lenses were applied in random order. They demonstrated that even mild myopic defocus significantly impaired field trial performance in dogs, leading to slower retrieval times and lower subjective evaluation scores [21].

Several studies have provided evidence that myopia is associated with structural changes in the brain, particularly in the visual cortex. Wu et al. reported a reduction in cortical thickness in individuals with myopia, suggesting altered cortical development or atrophy linked to refractive error [22]. Similarly, Huang et al. found a decrease in gray matter volume in visual processing areas, further supporting the notion of neuroanatomical alterations associated with myopia [23]. Additionally, Takeuchi et al. documented reduced overall intracranial volume in myopic subjects, indicating that myopia may be linked not only to localized cortical changes but also to broader brain structural differences [24]. These findings highlight the need for further research in veterinary medicine to better understand whether similar neuroanatomical changes occur in myopic animals and how they might affect behavior and visual function.

A recent report of recessively inherited CSNB in Beagles describes dogs affected with night blindness and a normal-appearing fundus, but ERG showed an absence of the dark-adapted b-wave with a normal a-wave [2]. Unlike Beagles with CSNB, the ERG results for the Beagles in this study were normal. However, in one Beagle (D6), a- and b-wave amplitudes were partially reduced, most likely due to incorrect electrode placement or incomplete mydriasis. Electroretinography was not repeated due to the significantly longer anesthesia time. Despite the unlikelihood of the presence of CSNB, a genetic test for the LRIT-3 gene was initiated on the six animals. None of the dogs tested were found to carry the LRIT3 gene mutation.

## 5. Conclusions

In dogs with visual impairment at night, retinoscopy should be performed in addition to the ophthalmological and ERG examination to rule out myopia as a potential cause. Further studies are needed to investigate the cause of myopia in the tested Beagle population and to explore any possible genetic factors.

## Figures and Tables

**Table 1:** Age, maze test results under dim light, and refractive values of 12 Beagle dogs.

Dog	Age	Maze Test in Dim Light			Refractive Values	
		Successful	Cautious	Failed	OD	OS
A1 *	16 y		x		−1.25	−1.25
B1 *	11 y			x	−4	−4.25
B2 *	11 y	x			−0.5	−0.5
C1 *	9 y			x	−6	−6.25
C2 *	9 y			x	−5.5	−5.5
D1	6 y		x		−1.75	−1.5
D2 *	6 y		x		−2	−2.25
D3	6 y	x			−1.25	−1.25
D4	6 y			x	−1.5	−1.5
D5	6 y			x	−3.5	−3.75
D6	6 y		x		−5.5	−5.5
D7	6 y	x			−0.5	−0.25
**Mean refractive values**		0.71	2.63	4.18		

* Dogs showing incipient cataracts and/or nuclear sclerosis in the ophthalmologic examination. y = years, x = observed performance in the dim light maze test. OD = oculus dexter (right eye), OS = oculus sinister (left eye).

**Table 2:** Measurements of eye parameters in nine dogs using B-scan ultrasound.

Dog	Axial Length (mm)		Cornea-Lens (mm)		Lens (mm)		Lens-Retina (mm)	
	OD	OS	OD	OS	OD	OS	OD	OS
B1	21.4	21.5	3.9	3.8	7.5	7.6	10.0	10.1
B2 *	20.9	20.8	3.8	3.9	7.6	7.5	9.5	9.4
D1	21.3	21.4	3.7	3.6	7.6	7.6	10.0	10.2
D2	21.6	21.5	3.7	3.8	7.6	7.5	10.3	10.2
D3	20.9	21.0	3.7	3.7	7.4	7.3	9.8	10.0
D4	21.6	21.5	3.8	3.8	7.5	7.6	10.3	10.1
D5	20.9	21.1	3.3	3.8	7.5	7.6	9.7	9.7
D6	21.3	21.6	3.8	3.8	7.5	7.5	10.0	10.3
D7 *	20.5	20.4	3.3	3.3	7.6	7.6	9.6	9.5

* Dogs with emmetropia (−0.25 to −0.5 diopters). OD = oculus dexter (right eye), OS = oculus sinister (left eye).

**Table 3:** Electroretinogram measurements of four dogs, showing the mean values for the right and left eyes of each individual. The values are given in microvolts (µV) and represent the average response of the retina to the light stimulus.

	Dark-Adapted ERG				Light-Adapted ERG		
Dog	Flash 3.0 cd·s/m^2^		Flash 10.0 cd·s/m^2^		Flash 3.0 cd·s/m^2^		Flicker 3.0 cd·s/m^2^
	a-Wave µV	b-Wave µV	a-Wave µV	b-Wave µV	a-Wave µV	b-Wave µV	µV
B1	92.5	191.5	131.5	212.5	7.7	33.4	46.7
D1	44.7	180.5	68.4	195.0	1.4	16.3	15.0
D5	71.8	184.5	104.7	218.5	7.0	31.8	53.6
D6	16.7	124	44.5	121.0	3.7	7.8	20.5
Mean	56.4	170.1	87.3	186.8	4.9	22.3	33.9

ERG = electroretinogram, cd·s/m^2^ = candela seconds per square meter, µV = microvolt.

## Data Availability

The raw data supporting the conclusions of this article will be made available by the authors on request.
